# Country readiness and prerequisites for successful design and transition to implementation of essential packages of health services: experience from six countries

**DOI:** 10.1136/bmjgh-2022-010720

**Published:** 2023-01-18

**Authors:** Ala Alwan, Reza Majdzadeh, Gavin Yamey, Karl Blanchet, Alemayehu Hailu, Mohamed Jama, Kjell Arne Johansson, Mohammed Yusuf Ahmed Musa, Omar Mwalim, Ole Frithjof Norheim, Najibullah Safi, Sameen Siddiqi, Raza Zaidi

**Affiliations:** 1 DCP3 Country Translation Project, London School of Hygiene & Tropical Medicine, London, UK; 2 School of Health and Social Care, University of Essex, Colchester, Essex, UK; 3 Center for Policy Impact in Global Health, Duke Global Health Institute, Duke University, Durham, North Carolina, USA; 4 Geneva Centre of Humanitarian Studies, University of Geneva, Geneva, Switzerland; 5 Bergen Center for Ethics and Priority Setting, Department of Global Public Health and Primary Care, University of Bergen, Bergen, Norway; 6 Harvard University T H Chan School of Public Health, Boston, Massachusetts, USA; 7 Ministry of Health, Federal Government of Somalia, Mogadishu, Somalia; 8 Health System Development, Health Financing, WHO Country Office for Sudan, Khartoum, Sudan; 9 WHO Country Office for Afghanistan, Kabul, Afghanistan; 10 Department of Community Health Sciences, Aga Khan University Medical College Pakistan, Karachi, Sindh, Pakistan; 11 Pakistan Ministry of National Health Services, Regulations, and Coordination, Islamabad, Pakistan

**Keywords:** Health policies and all other topics, Health services research, Health systems, Health economics

## Abstract

This paper reviews the experience of six low-income and lower middle-income countries in setting their own essential packages of health services (EPHS), with the purpose of identifying the key requirements for the successful design and transition to implementation of the packages in the context of accelerating progress towards universal health coverage (UHC). The analysis is based on input from three meetings of a knowledge network established by the Disease Control Priorities 3 Country Translation Project and working groups, supplemented by a survey of participating countries.

All countries endorsed the Sustainable Development Goals target 3.8 on UHC for achievement by 2030. The assessment of country experiences found that health system strengthening and mobilising and sustaining health financing are major challenges. EPHS implementation is more likely when health system gaps are addressed and when there are realistic and sustainable financing prospects. However, health system assessments were inadequate and the government planning and finance sectors were not consistently engaged in setting the EPHS in most of the countries studied. There was also a need for greater engagement with community and civil society representatives, academia and the private sector in package design. Leadership and reinforcement of technical and managerial capacity are critical in the transition from EPHS design to sustained implementation, as are strong human resources and country ownership of the process. Political commitment beyond the health sector is key, particularly commitment from parliamentarians and policymakers in the planning and finance sectors. National ownership, institutionalisation of technical and managerial capacity and reinforcing human resources are critical for success.

The review concludes that four prerequisites are crucial for a successful EPHS: (1) sustained high-level commitment, (2) sustainable financing, (3) health system readiness, and (4) institutionalisation.

**Summary box**

- Setting and revising essential packages of health services must be country-executed and country-owned.
- Packages developed without sustained commitment from high level government leaders and adequate engagement of national authorities are less likely to be implemented.
- Early, committed engagement of the government’s planning and finance sectors is essential in ensuring affordability and committed funding of the universal health coverage (UHC) package—there is limited value in investing in package development without a realistic financing plan.
- Even a perfectly designed, affordable package has no major impact unless health system gaps are addressed and there are adequate and well-trained human resources to deliver effective services—including a clear role for the private sector.
- Sustainability for revising and implementing UHC packages requires leadership, political stability, sustained resources and institutionalisation of technical and managerial capacity.
- There is a need to reinforce technical assistance to low-income and lower middle-income countries in UHC-related programmes, including through regional institutions.

## Introduction

Although countries pledged to achieve universal health coverage (UHC) by 2030,^1^ the current pace of progress indicates that more than one-third of the world’s population will not have access to essential health services by this target date.^2^ The situation is compounded by the adverse effects of the COVID-19 pandemic on health systems worldwide and the resulting disruptions in access to quality health services. Since countries must re-double their efforts in improving such access, there is growing demand from governments of low-income and lower middle-income countries (LLMICs) for technical assistance in health system strengthening and UHC.^3^

The third edition of the Disease Control Priorities series (DCP3)^4 5^ provides an up-to-date review of cost-effectiveness of health interventions through a systematic appraisal of evidence, new economic analysis and expert judgement of a wide range of health services. The goal is to influence resource allocation decisions at country level to achieve the highest impact of health interventions provided by LLMICs.

Two model essential packages of health services (EPHS)—also called health benefits packages—were developed to serve as a guide and starting point for the development or revision of EPHS. The first is the essential UHC (EUHC) package, which includes 218 interventions designed for lower middle-income countries. The second is the high priority package, a subset of the EUHC, that includes 108 interventions, proposed as a model for low-income countries. The criteria adopted for selecting health services are evidence of impact, cost-effectiveness, financial risk protection, equity and feasibility of implementation.^4^ The DCP3 approach recommends that the package is financed publicly and is implemented to achieve UHC in a stepwise manner through a progressive universalism approach.^5^ In this approach, the package is initially designed to provide highly cost-effective health services, particularly for diseases that disproportionately affect the poor. As health resources grow, coverage will increase and include a wider range of interventions.^6^ By publicly financing the highest priority health services, the DCP3 approach covers the three key dimensions of UHC: providing unimpeded access to all population groups, expanding the range of essential services and reducing financial risk.

Since their launch in December 2017, the DCP3 evidence and packages have been used by several countries to design or revise their national EPHS.^7–10^ The experience of six LLMICs has recently been reviewed by a knowledge network of professionals engaged in DCP3-related country work to extract lessons learnt and update evidence and good practice. The review covered seven key areas in the process of designing an EPHS: requirements for successful EPHS design (current paper), decision-making processes,^11^ estimating costs,^12^ financing,^13^ building implementable packages, role of the private sector^14^ and monitoring and evaluation.^15^ The seven papers in this collection cover the key findings of the review. Because there is limited value in investing in the development of an EPHS if the process does not lead to high-level government endorsement, this first paper specifically assesses the requirements for an appropriate design of UHC packages. Since the ultimate goal is to improve healthcare, the paper also aims to identify the essential elements needed for the transition from package design to implementation and improved access to services that are essential for accelerating progress to UHC.

We conducted an initial review of the experiences of Afghanistan, Ethiopia, Pakistan, Somalia, Sudan and Zanzibar–Tanzania in setting their own EPHS by establishing a knowledge network of professionals working in priority setting and UHC-related policies. The World Bank classifies Pakistan and Zanzibar–Tanzania as lower middle-income countries, and the remaining four as low-income countries.^16^ Key representatives from each of the six countries presented their experiences during the first DCP3 country review meeting in Geneva on 27–28 September 2021, organised by the DCP3 Country Translation Project at the London School of Hygiene & Tropical Medicine. It was attended by a network of 60 experts and professionals engaged in DCP3-related work. The team collectively decided to conduct a survey to fill in gaps and standardise data from all six countries and to stimulate group discussions. A group consisting of the authors of this paper updated and presented the review to the network during the second and third DCP3 country review meetings held on 6 December 2021 and 31 March 2022, respectively. The current analysis is based on the discussions in these meetings and the group work in between meetings. Online supplemental box S1 summarises the review meeting, the survey and the development of the framework for this paper.

10.1136/bmjgh-2022-010720.supp1 Supplementary data

Box 1 **An outline of requirements for country readiness and prerequisites for successful design of essential packages of health services and transition to implementation**

*Securing political commitment*

1. Ensuring sustained political commitment for universal health coverage (UHC).
  - Commitment and a clear government position, including that of financing and planning sectors.
  - Commitment at the level of the parliament.
  - Commitment at the subnational level, particularly in decentralised systems.
  - Demonstrated commitment to fund the package and to finance the UHC road map.

*Engaging key stakeholders*

1. Conducting stakeholder analysis of key national players, including the private sector, academic and public health institutions, community representatives and external partners.
2. Engaging the planning and finance government sectors and the National Bureau of Statistics as early as possible.
3. Building national consensus and conducting societal dialogue on health services.

*Assessing health system and financing mechanisms*

1. Conducting an in-depth assessment of the health system, including governance structure, infrastructure, delivery arrangements, health workforce, information system.
2. Mapping of health services currently provided, based on the DCP3 model packages, UHC Compendium or previously existing packages.
3. Assessing fiscal space, existing health financing mechanisms and sustainability of health financing; deciding on the level of public funds provided to finance the package.

*Developing and implementing a road map*

1. Agreeing on principles, especially transparency, impartiality and inclusiveness.
2. Defining a governance structure to design the EPHS and sustain implementation and revision.
3. Ensuring prioritisation and package costing are data-driven and evidence-informed.
4. Agreeing on the steps, decision criteria and processes for prioritising and costing interventions.
5. Defining the scope of the EPHS, including health delivery platforms with special focus on primary healthcare.
6. Developing an action plan, including roles, mandates, required skills and resources, including capacity building in priority setting and UHC package design.

*Securing a successful transition to sustainable implementation*

1. Ensuring affordable and sustainable financing of high-priority health services along the UHC timeline.
2. Addressing health system gaps and reinforcing health service delivery, including the role of the private health sector.
3. Addressing the risk of instability in fragile and politically unstable contexts and proposing risk mitigation measures with stakeholders.

## Package design process

The other papers in this collection describe the different processes followed by the six countries to design their packages.^11–15^ In this paper, we focus on steps that directly relate to the requirements that determine successful package design and increase the prospects of a successful rollout.

All countries required 1–3 years to design their packages (table 1). This is not surprising given the importance of ensuring that the guiding principles are adopted, effective partnerships are secured, baseline assessments are effectively conducted and processes for prioritisation and costing are sound and reliable. The time taken to develop the package also depends on local circumstances and capacity and varies considerably between countries. The Pakistan and Sudan packages were developed for the first time, while others were revisions and expansions of earlier versions that existed before the endorsement of the Sustainable Development Goals (SDG) target 3.8 in 2015. Baltussen *et al* review the experience of the six countries in prioritising essential health services.^11^ All countries used the DCP3 evidence to guide the prioritisation of essential health services, but two countries, Somalia and Sudan, used additional sources of evidence such as the WHO–UHC Compendium.^17^ Packages covered all DCP3 delivery platforms (community, health centre, first-level hospital, tertiary care and population-based interventions), but the Pakistani government decided early in the course of the package design to focus during the first 5 years of implementation on the district level system, which covers the first three platforms.

**Table 1:** Background information on and characteristics of the EPHS in the six focus countries

	Afghanistan	Ethiopia	Pakistan	Somalia	Sudan	Zanzibar (Tanzania)
Year of completion of the package	2021	2019	2020 (Generic EPHS), 2021 (six provincial and federating areas EPHS)	2020	2022	2022
Time required to construct the package	2 years	1–2 years	2 years	1–2 years	2 years	3 years
Main source of evidence adopted as a guide	DCP3	DCP3 expanded with service listing in UHC Compendium	DCP3	DCP3 and UHC Compendium	DCP3 expanded with other packages	DCP3
New package or revision based on a previous package	Revision and expansion	Revision and expansion	New package*	Revision and expansion	New package	Revision and updating
Delivery platforms targeted by the package	All delivery platforms†	All delivery platforms	District-level platforms‡	All delivery platforms	All delivery platforms	All delivery platforms
Cost of the EPHS in US$ per capita in the first year§	6.9	40.0	13.0 for the federal EPHS¶	7.4	23.3	37.0
Number of interventions in the final package	158	1018	88	412	824	314
Position of the country on the source of financing the final package	Prepayment schemes and donor funding	Public finance, donor funding and user fees	Public finance with gap filling from donor funding	Public finance, donor funding and user fees	Public finance, prepayment, donor funding and user fees	Public finance, public health insurance, prepayment, donor funding and user fees

*A rudimentary EPHS existed in two provinces focusing on a few programmes.

†DCP3 delivery platforms are community, health centre, first level hospital, referral and specialty hospitals and population-based interventions.

‡All five delivery platforms were prioritised and costed, but the government decided to initially implement the three district level platforms.

§All packages will have increasing coverage along the timeline of the Sustainable Development Goals target 3.8 and the projected costs will be considerably higher and require an increase in health allocation.

¶EPHS in each province/area has a different set of interventions and unit cost.

DCP3, third edition of the Disease Control Priorities series; EPHS, essential packages of health services; UHC, universal health coverage.

Conducting an analysis of the fiscal space and determining how the UHC services will be funded is a critical step at the onset. The decision on how the package is financed during the implementation phase varied between countries. Based on their national vision and strategic plan, policymakers in Pakistan decided on public financing of the 88 high-priority services of the district-level package. The other five countries adopted financing mechanisms that included other prepayment schemes, including health insurance schemes, combined with user fees and donor funding.

The cost of the package also varied (table 1). Gaudin *et al* reviews the costing process conducted in five of these countries.^12^ Afghanistan had the lowest per capita package cost in the first year of implementation (US$6.9), while Ethiopia had the highest (US$40). In some cases, the per capita cost during initial implementation was higher than the public health expenditure or even the current health expenditure. For the first year of implementation, this situation required either further prioritisation or consideration of different financing schemes for the different groups of interventions.

Because of the differences in the delivery platforms and in financing mechanisms, the number of health interventions varied considerably, from 88 in Pakistan to over 1000 in Ethiopia.

## Securing political commitment

Political commitment to UHC and the resolve to improve access to essential health services is one of the key prerequisites to reaching SDG target 3.8; we analysed such commitment through four questions (online supplemental table S1). Since there is no reliable tool to accurately assess political commitment, these questions were considered as proxy indicators. All six countries endorsed UHC as a target in their national health strategies and the UHC package is part of the national SDG plans and monitoring schemes. However, the planning and finance sectors of the government have not been consistently engaged in most countries; despite participation in meetings and discussions, their engagement has not been sustained in some countries, particularly in the fiscal space assessment and the planning for increased funding. There was also no evidence of a concrete commitment by parliament.

10.1136/bmjgh-2022-010720.supp2 Supplementary data

## Assessing health system and financing mechanisms

Assessment of health system performance and capacity, including financing, is a critical step at the onset, since its outcome has a major influence on package design, and how it will be financed and implemented. Table 2 presents the most recently reported information on selected health system indicators, particularly those focusing on the status of health services delivered, health financing and the health workforce.

**Table 2:** Selected health system indicators for the EPHS*

	Afghanistan	Ethiopia	Pakistan	Somalia	Sudan	Zanzibar† (Tanzania)
UHC Service Coverage Index	37	39	49.9 (2020)	25	44	43*
Proportion of population with household expenditures on health greater than 10% of total household expenditure or income (SDG 3.8.2) (%)	14.6 (2013)	4.9 (2015)	4 (2017)	NA	18.4 (2009)	3.8 (2011)*
Current health expenditure (CHE) per capita (US$)	66	33	48	NA	47	34
Domestic general government health expenditure (GGHE-D) as percentage of gross domestic product (%)	1.08	0.74	1.08	NA	1.04	1.56*
Government spending for health per capita US$	5	6	19	NA	11	18.2
Ratio between the EPHS cost per capita in the first year of implementation and government spending for health	1.4	6.7	0.7	NA	2.1	2.0
GGHE-D as percentage of general government expenditure (%)	3.9	4.8	6.0	2.0	5.6	9.5
Out-of-pocket expenditure as percentage of CHE (%)	79.3	37.9	56.5	NA	67.4	16.0
Medical doctor per 10 000 population	2.5	1.1	11.2	0.22	2.6	0.5*
Nursing and midwifery personnel per 10 000 population	4.5	7.8	4.8	1.2	11.5	5.7*

*Data included in this table are mainly based on the WHO’s Global Health Observatory, except for figures updated or reported by the country representatives. https://www.who.int/data/gho/data/indicators. Last accessed on March 19, 2022.

†Data highlighted with an asterisk (*) belong to Tanzania; the rest is for Zanzibar.

EPHS, essential packages of health services; NA, not available; SDG, Sustainable Development Goals; UHC, universal health coverage.

The UHC service coverage index is below 50 in all countries (on a scale from 0 to 100, based on 16 tracer indicators of health service coverage), with a range between 25 in Somalia and 49.9 in Pakistan.

A major challenge in financing the EPHS is that the fiscal space for public expenditure on health is very limited in all countries and it is considerably lower than the initial cost required for implementing the package in some of these countries. The current per capita health expenditure ranged between US$33 in Ethiopia and US$66 in Afghanistan. Per capita public spending on health is as low as US$5 and US$6 in Afghanistan and Ethiopia, respectively. Government spending on health as a percentage of the current health expenditure ranges from less than 10% in Afghanistan to 53% in Zanzibar. Out-of-pocket expenditure as a share of current health expenditure is high in most countries, with the highest being in Afghanistan (79%). The incidence of catastrophic expenditure (household spending on health that exceeds 10% of total household budget) is lowest in Zanzibar and highest in Sudan.

Compared with the financial resources available in the six countries, the cost of the package illustrated in table 1 is higher than the government health expenditure in some of them. Table 2 presents the ratio of the package cost to the government health expenditure, which ranges between 0.7 and 6.7. The mismatch between the financial resources available for public spending and the estimated cost of the package represents a major challenge for the transition to implementation, even if part of the financing gap is covered by development partners and donors.

An equally important challenge of the transition to effective implementation is the health system capacity in LLMICs. Although all health system building blocks need to be assessed, the state of human resources is especially important since it often impedes access to health services and influences the prioritisation of interventions and the overall design of the essential package. Based on the SDG index threshold of doctors, nurses and midwives,^18^ the figures in table 2 indicate serious shortages in the healthcare workforce in the countries reviewed.

## Engaging key stakeholders

A comprehensive stakeholder analysis was not conducted in some countries at the initial stage of package development (table 3).

**Table 3:** Stakeholders’ involvement in the six countries

	Afghanistan	Ethiopia	Pakistan	Somalia	Sudan	Zanzibar (Tanzania)
Was any stakeholders’ analysis conducted at the initial stage of the UHC EPHS development?	No	No	Yes	Yes	No	No
Who were the national and international stakeholders centrally involved in the EPHS process?						
Parliament	No	No	Some parliamentarians	Social Standing Committee	No	No
Finance	No	Director of Economic Planning	Yes	No	No	Yes
Planning	No	No	Member of Social Sector of Planning Commission and staff	Yes	No	Yes
Community and patients’ groups	No	No	No	No	No	Yes
Private sector	No	No	Yes	Yes	No	No
National academia	No	National universities	Aga Khan University and Health Services Academy	Yes	National Public Health Institute	No
Multilateral organisations	WHO	WHO, UNICEF, UNFPA, WFP IOM and UN health and nutrition cluster	WHO, UNICEF, Global Fund, GAVI and WB	WHO, UNICEF UNFPA and WB	WHO	WHO and UNICEF
Humanitarian and development partners	BMGF and WB	UK, Italy, USAID, Sweden, Switzerland, Norway, Finland, Germany and Canada	BMGF, USAID and FCDO	UK, Sweden, Italy, Switzerland, Canada, Germany, USAID, Gavi and Global Fund	EU	WHO, UNICEF and UNFPA
Others	International academic institutes	International academic institutes	International academic institutes	–	Consultancy firm	International academic institutes and Bureau of statistics
What type of support was provided by multilateral organisations?						
WHO	Technical support	Technical and financial support	Technical and financial support	Technical and financial support	Technical support	Technical and financial support
UNICEF	Technical support	Technical review	Technical and financial support	Technical support	No	Technical support
UNDP	All no					
World Bank	Technical support	Technical and financial support	Technical and financial support	Technical and financial support	Technical support	No

*An existing profile from Reform Directorate at the MOH was used.

BMGF, Bill & Melinda Gates Foundation; EPHS, essential packages of health services; EU, European Union; FDCO, Foreign, Commonwealth and Development Office UK; GAVI, GAVI, the Vaccine Alliance; IOM, International Organization for Migration; MOH, Ministry of Health; UHC, universal health coverage; UNFPA, United Nations Population Fund; UNICEF, United Nations International Children's Emergency Fund; USAID, United States Agency for International Development; WB, World Bank; WFP, World Food Programme.

As highlighted before, not all countries had meaningful engagement of the finance and planning sectors, and only one country engaged community and civil society representatives during the package design process (table 3). The private health sector, which currently delivers a major proportion of the high-priority health services,^14 19^ did not seem to be involved in four out of the six countries. A more elaborate review of the role of the private sector in package design and implementation is covered by Siddiqi *et al*.^14^ Of the multilateral partners, the WHO was involved in providing technical assistance in all countries and the United Nations International Children’s Emergency Fund (UNICEF) and the World Bank in most of them. However, the United Nations Development Programme (UNDP) did not seem to be significantly engaged in all six countries.

## Developing and implementing a road map

Table 4 includes findings on the package development process and the various elements of the road map. At least two countries do not have a unit on priority setting, health economics or health technology assessment—such a unit is needed for designing, revising and monitoring the UHC package. Institutionalisation and capacity strengthening in these areas within the Ministry of Health varied between countries. The list of priority decision-making areas where training and capacity building is required covers priority setting, health technology assessment, equity analysis, costing, budget impact and health service delivery models.

**Table 4:** Essential elements of a road map for EPHS development in the six focus countries

	Afghanistan	Ethiopia	Pakistan	Somalia	Sudan	Zanzibar (Tanzania)
Establishing a project secretariat within the MOH	Yes	Yes	Yes	Yes	No	Yes
Existence of a unit within the MOH for health economics or health financing	Yes	Yes	Yes	No	Yes	No
Considering capacity building in the initial action plan for developing UHC HBP	Yes	Yes	Yes	No	No	Yes
Identifying and acting on areas required for training and capacity building	Priority setting, health system assessment and health economics	Systematic review, economic evaluation, equity analysis, budget impact and costing	Evidence generation and use in prioritisation, organisational restructuring for the implementation, management and health systems	Burden of disease, health system performance assessment, health economics, economic evaluation, equity analysis, budget impact and costing	Priority setting	Priority setting, health economics and costing
Capacity building mechanisms that have been adopted/implemented	On-job training	Training and graduate studies at MSc and PhD Level	Training, inside and outside of the country and graduate studies	No	Short trainings	Short trainings for 12 members of core team and 1 Master and 2 PhDs
Institutionalising capacity building in the plan	No	Yes	To some extent	No	Yes	No
Developing a communication strategy as part of the EPHS action plan	No	No	Yes, during implementation	No	No	No

EPHS, essential packages of health services; MOH, Ministry of Health; UHC, universal health coverage.

## Key challenges and lessons learned

The review highlights critical challenges at different stages of the EPHS development. Some of these challenges are directly related to the processes and methodologies used, including gaps in preparation and in readiness, while others are inherent in the capacity, resources and performance of the health system. These challenges were compounded by the timing of the exercise in some countries that coincided with the COVID-19 pandemic.

UHC is a political choice and priority.^1^ Only two countries reported some engagement of parliamentarians at some stage of the package development process, but there were no formal decisions or resolutions made on UHC or the EPHS. Similarly, although most countries reported some involvement of the finance and planning sectors in the package development process, the level and timing of engagement was not clear.

As mentioned before, assessing health systems building blocks and financing mechanisms is an essential component of package development. Scarcity of health resources requires trade-offs and tough choices. An extreme case is the situation in Afghanistan, where only about 8% of the current health expenditure is financed through the government budget and almost 80% comes from out-of-pocket expenditure. The countries reviewed have low levels of domestic government health expenditure (table 2), which are very far from the Abuja Declaration target of spending 15% of government budgets on health.^20^ As noted in the findings, funding the package should not only cover initial implementation. Population growth in some countries and the progressive increase in the coverage of interventions along the UHC timeline will result in rising costs that will far exceed the current expenditure and any forecasted economic growth. Therefore, between initial implementation and 2030 UHC targets, governments must plan for additional resources.

The experience in the six countries indicated that all of them must consider feasible options for increased health allocations, including re-prioritisation of the government budget. Irrespective of the level of fiscal constraints, the focus on public financing must be on high-priority, high-impact interventions.^5^ Overall, implementation is less likely if the country invests in a package development exercise without first assuring realistic and sustained finance prospects. Soucat *et al* in this series review the challenges, current experience and directions on package financing.^13^

Countries also have limited capacity because of serious gaps across health system building blocks. In such a situation, two difficult questions should be addressed by policymakers in countries aiming to achieve UHC.^21^ First, which health services should be considered for full population and full cost coverage? Second, how can health planners expand the size or power of pooled funds so that they can achieve UHC? Addressing these questions should be at the centre of the health system assessment that is conducted at the initial phase and in parallel with the prioritisation phase.

It is not possible to deliver effective services without the necessary human resources. Addressing these gaps through a carefully planned and realistic health workforce strategy will be a basic requirement for the transition from package design to implementation.^22^ Capacity building should start as soon as possible based on the health service delivery model required for package implementation.

Engaging key internal and external stakeholders is critical for national ownership of the process and its outcome. In this respect, we highlight the importance of civil society dialogue and engagement of community groups particularly in focusing on people’s perceptions of their priority health needs.^23^ Apart from Zanzibar, where this requirement has been partly acknowledged, none of the countries were engaged in serious interaction with people’s representatives. Thailand’s experiences in implementing UHC and forming the National Health Assembly for public participation is worth highlighting. The commitment to UHC became a collective community demand. As a result, despite an unstable political environment, repeated changes of governments and ministers of health and frequent elections, the commitment to achieve the required change and implement the package was maintained.^24^ In contrast, package implementation in Colombia, the Dominican Republic and Peru has faced significant challenges since national stakeholders and especially the beneficiaries were not involved in the package development.^25^

The role of external stakeholders is equally important. Experience indicates that technical and logistic support from WHO and UNICEF has been invaluable. However, as previously mentioned, it is important to note that UNDP, which coordinates the SDG monitoring initiatives, has not been significantly involved. In general, no strong coordination appears to have been established with the SDG monitoring part of the government.

Although all six countries reviewed in this paper illustrated an initial political commitment to the package (online supplemental table S1), sustainability of this commitment in countries with political instability is a major concern. Afghanistan, Somalia, Pakistan, Sudan and Ethiopia rank highly among almost 200 countries globally for likelihood of political instability and/or politically-motivated violence, including terrorism.^26^ After defining the package, Afghanistan faced a regime change, and Pakistan, Sudan and Somalia had changes in government. Such instability is commonly associated with economic constraints and limited fiscal space for health and social services.

Sustainability of EPHS implementation is more likely if economic realities are seriously considered during the package design and the technical expertise and skills are institutionalised.^27^ The dynamic of power and financing are important in package design and implementation because these often involve trade-offs and redistribution of resources between services and service providers. Good governance is therefore needed to ensure that setting EPHS follows equity and fairness principles, including stakeholders’ involvement, transparency and accountability, particularly in defining entitlements and identifying benefits. This is also why institutionalisation needs a strong leadership structure that meets legal, political, technical, human and financial requirements to govern the EPHS at the national or subnational level, depending on the authority structure of the country.^28^ Countries will be moving in the right direction if the governance structure is adopted or adapted to serve as a permanent UHC or national health reform and coordination unit. In this respect, capacity building is critical and sustainable skills are essential for priority setting, monitoring and evaluation and periodic revision of interventions.

## Basic requirements for successful package design and transition to implementation

Many countries designed EPHS before setting UHC as a global commitment in 2015,^25 29 30^ but not all were implemented. Some packages were defined by countries in fragile situations and chronic crises, sometimes with the support and engagement of development agencies and donors, such as in Afghanistan,^7^ northern Syria,^31^ Somalia^32^ and Yemen.^33^ In some of these countries, the package aimed to identify a minimum set of essential services for emergency funding or for contracting with the private sector or non-governmental organisations to provide primary care services. While these reasons justify the need for the service package, the definition of the EPHS in the six countries has a broader objective and serves as a major milestone for the realisation of UHC. It is with this wider scope in mind that we reviewed the experience of six LLMICs in preparing for the development of the package and ensuring a robust process and a successful outcome. Based on the experience of these countries, we believe that the steps listed in box 1 are necessary for a successful design and transition to implementation of the EPHS.

We believe that all steps in this box are important in reinforcing the EPHS development process, particularly those that address gaps impeding implementation. As stated previously, there are key requirements that have to be considered in order to address the limitations in the package development process highlighted by the review. First, strong decisions on package rollout are more difficult to make in the absence of high-level government leadership and ownership, as well as active engagement of key stakeholders. Second, it is not realistic to expect improved access to essential interventions without a serious assessment of financing needs and health system constraints and related plans to secure the required resources and to address existing health system gaps. Third, experience shows that the private sector does not play a significant role in the design of UHC packages despite the fact that it currently provides a major proportion of primary healthcare services in the six countries reviewed. It is essential to consider measures to address key barriers related to governance, regulation, accountability and quality of services. The fourth requirement is that of institutionalisation. Priority setting, package design and monitoring of implementation are part of a dynamic process which requires building managerial and technical capacity in ministries of health and partner institutions in order to respond to policy changes and to monitor implementation. In this respect, we also believe that establishing and strengthening existing regional centres will make an important contribution to national efforts to institutionalise package design and implementation capacities in LLMICs.

In general, the analysis of the experience in the six countries demonstrates areas of strengths and weaknesses. We believe that a review of the contents of box 1 will support countries in identifying areas where reinforcement is needed during package design or revision.

## Conclusions

Based on the experience of the six countries, we believe that the steps listed in box 1 are necessary for successful design and transition to implementation of a UHC package of essential health interventions. Two categories of steps are related to initial situation analysis, including the level of political commitment and the state of the country’s health system. The remaining steps focus on other components of the package development process and transition to implementation. Despite the achievements made by all countries and the innovation involved, the review suggests important areas that warrant further strengthening, particularly in relation to the requirements necessary for the transition from package design to implementation. These areas include the need for stronger engagement of key government stakeholders, involvement of civil society and community groups, an in-depth review of the health system, including health financing mechanisms and service delivery models, and ensuring the engagement of the private sector. It is also essential to reinforce technical support to LLMICs in these areas.

## Data Availability

All data relevant to the study are included in the article or uploaded as supplementary information.

## References

[1] Political declaration of the high-level meeting on universal health coverage. Global health and foreign policy. New York: United Nations General Assembly, 2019. https://www.un.org/pga/73/wp-content/uploads/sites/53/2019/07/FINAL-draft-UHC-Political-Declaration.pdf

[2] World Health Organization, World Bank. Tracking universal health coverage: 2017 global monitoring report. Geneva World Health Organization; 2017.

[3] World Health Organization. Who presence in countries, territories and areas: 2021 report. Geneva: World Health Organization, 2021.

[4] Volumes DCP3. Available: http://dcp-3.org/volumes [Accessed 08 Jul 2022].

[5] Jamison DT, Alwan A, Mock CN, et al. Universal health coverage and intersectoral action for health: key messages from disease control priorities, 3rd edition. Lancet 2018;391:1108–20. 10.1016/S0140-6736(17)32906-9 29179954 PMC5996988

[6] Jamison DT, Summers LH, Alleyne G, et al. Global health 2035: a world converging within a generation. Lancet 2013;382:1898–955. 10.1016/S0140-6736(13)62105-4 24309475

[7] Blanchet K, Ferozuddin F, Naeem AJJ, et al. Priority setting in a context of insecurity, epidemiological transition and low financial risk protection, Afghanistan. Bull World Health Organ 2019;97:374–6. 10.2471/BLT.18.218941 31551635 PMC6747028

[8] Eregata GT, Hailu A, Geletu ZA, et al. Revision of the Ethiopian essential health service package: an Explication of the process and methods used. Health Syst Reform 2020;6:e1829313. 10.1080/23288604.2020.1829313 33300838

[9] Ministry of National Health Services, Regulations and Coordination, Disease Control Priorities, World Health Organization. Universal Health Coverage Benefit Package of Pakistan: Essential Package of Health Services with localized evidence. MoNHSR&C: Islamabad; 2020. http://dcp-3.org/resources/universal-health-coverage-benefit-package-pakistan-essential-package-health-services [Accessed 12 Sept 2022].

[10] Ministry of Health & Human Services. Federal Republic of Somalia. the essential package of health services (EPHS) Somalia 2020, 2021. Available: https://reliefweb.int/report/somalia/essential-package-health-services-ephs-somalia-2020 [Accessed 12 Sept 2022].

[11] Baltussen R, Mwalim O, Blanchet K, et al. Decision-making processes for essential packages of health services: experience from six countries. BMJ Glob Health 2023;8 10.1136/bmjgh-2022-010704 PMC9853142 36657809

[12] Gaudin S, Raza W, Skordis J, et al. Using costing to facilitate policy making toward UHC: findings and recommendations from country-level experiences. BMJ Glob Health 2023;8:e10735. 10.1136/bmjgh-2022-010735 PMC9853124 36657806

[13] Soucat A, Tandon A, González-Pier E. From UHC benefit packages to budget appropriation: the long journey to implementation. BMJ Glob Health 2023. 10.1136/bmjgh-2022-010755 PMC10186405 37188361

[14] Siddiqi S, Aftab W, Raman AV, et al. The role of the private sector in delivering essential packages of health services: lessons from country experiences. BMJ Glob Health 2023;8:e010742. 10.1136/bmjgh-2022-010742 PMC9853132 36657810

[15] Danforth K, Ahmad A, Blanchet K, et al. Monitoring and evaluating the implementation of essential packages of health services. BMJ Glob Health 2023. 10.1136/bmjgh-2022-010726 PMC10069525 36977532

[16] The world bank. world bank country and lending groups. Available: https://datahelpdesk.worldbank.org/knowledgebase/articles/906519-world-bank-country-and-lending-groups [Accessed 08 Jul 2022].

[17] World Health Organization. The UHC compendium: a global Repository of interventions for UHC. v1.2. Available: https://www.who.int/universal-health-coverage/compendium [Accessed 08 Jul 2022].

[18] World Health Organization. Health workforce requirements for universal health coverage and the sustainable development goals. Geneva: World Health Organization, 2016.

[19] Zaidi S, Riaz A, Thaver A. Role and contribution of private sector in moving towards universal health coverage in the eastern Mediterranean region. Aga Khan university, 2012. Available: https://ecommons.aku.edu/pakistan_fhs_mc_chs_chs/193/

[20] World Health Organization. The Abuja declaration: ten years on. World Health organization, 2011. Available: https://apps.who.int/iris/handle/10665/341162

[21] Alwan A, Jamison D, Hatefi A. Universal health coverage: health systems interventions and intersectoral policies. In: Making health systems work in low- and middle-income countries. Cambridge University Press, 2017.

[22] Gilles D, Buchan J, Sermeus W. Assessing future health workforce needs. Geneva: World Health Organization Regional Office for Europe, 2010.

[23] World Health Organization. Principles of health benefit packages. Geneva: World Health Organization, 2021.

[24] Tangcharoensathien V, Patcharanarumol W, Suwanwela W, et al. Defining the benefit package of Thailand universal coverage scheme: from pragmatism to sophistication. Int J Health Policy Manag 2020;9:133–7. 10.15171/ijhpm.2019.96 32331492 PMC7182149

[25] Glassman A, Giedion U, Smith PC. What’s in, what’s out? Designing benefits for universal health coverage. Washington DC: Center for Global Development, 2017.

[26] Metadata glossary. Available: https://databank.worldbank.org/metadataglossary/worldwide-governance-indicators/series/PV.EST#:~:text=Political%20Stability%20and%20Absence%20of%20Violence%2FTerrorism%20measures%20perceptions%20of,%2Dmotivated%20violence%2C%20including%20terrorism [Accessed 08 July 2022].

[27] Sajadi HS, Jama M, Majdzadeh R. Institutionalisation is a Vital Element for Fairness of Priority Setting in the Package Design if the Target is Universal Health Coverage. Comment on "Evidence-Informed Deliberative Processes for Health Benefits Package Design – Part II: A Practical Guide". Int J Health Policy Manag 2023:7544. 10.34172/ijhpm.2022.7544 PMC10125206 37579458

[28] Norheim OF. Disease control priorities third edition is published: a theory of change is needed for translating evidence to health policy. Int J Health Policy Manag 2018;7:771–7. 10.15171/ijhpm.2018.60 30316224 PMC6186484

[29] Shekh Mohamed I, Hepburn JS, Ekman B, et al. Inclusion of essential universal health coverage services in essential packages of health services: a review of 45 low- and lower- middle income countries. Health Syst Reform 2022;8:e2006587. 10.1080/23288604.2021.2006587 35060830

[30] Wright J, Holtz J. Essential packages of health services in 24 countries: findings from a cross-country analysis. Bethesda, MD: USAID, 2017.

[31] World Health Organization. The essential primary health care package in northern Syria. Gaziantep:World Health Organization/Health Cluster, 2016.

[32] UNICEF. Essential package of health services. Somaliland, 2009.

[33] Garber K, Fox C, Abdalla M, et al. Estimating access to health care in Yemen, a complex humanitarian emergency setting: a descriptive applied geospatial analysis. Lancet Glob Health 2020;8:e1435–43. 10.1016/S2214-109X(20)30359-4 33069304 PMC7561303

