# Supplementary material for: Country readiness and prerequisites for successful design and transition to implementation of essential packages of health services: experience from six countries
Source: BMJ Glob Health. 2023 Jan 18;8(Suppl 1):e010720. doi: 10.1136/bmjgh-2022-010720 (PMC9853149; doi:10.1136/bmjgh-2022-010720)
Supplement: Supplementary data [file bmjgh-2022-010720supp001.pdf]

BMJ Publishing Group Limited (BMJ) disclaims all liability and responsibility arising from any reliance placed on this supplemental material which has been supplied by the author(s)

Box S1: Development of this paper: country review meeting, survey, and framework for defining and implementing EPHS

The DCP3 country review meeting in Geneva on 27-28 September 2021 covered a snapshot of the status and governance of the health system in each of the six countries, fiscal space for health, processes and methodologies used in developing the package, including data and evidence used, stakeholders and partners involved, lessons learned and status of implementation. A summary of each country study was shared and reviewed among participants in the meeting and subsequently updated by the presenters.

The authors of this paper constituted a group of public health experts and country representatives engaged in DCP3-related work with the aim of appraising the key requirements for setting EPHS based on international experience and lessons learned in the six countries. The group developed a proposed list of 'best practice' priority actions required to prepare for the development of the UHC package and in ensuring country readiness and fulfilment of the prerequisites for successful design and implementation of UHC health services packages. Building on the list, a survey was conducted with representatives of the six countries to obtain more information on the processes and methodologies used in each country.

Feedback received from the respondents was incorporated to produce a framework for defining and implementing essential packages, covering key issues that need to be considered and the actions taken in setting or revising the packages. The framework has two domains: (1) a readiness assessment, which covers political commitment and health system considerations, and (2) prerequisites for successful implementation, including stakeholders' involvement and a road map for implementation.
