# Supplementary material for: Country readiness and prerequisites for successful design and transition to implementation of essential packages of health services: experience from six countries
Source: BMJ Glob Health. 2023 Jan 18;8(Suppl 1):e010720. doi: 10.1136/bmjgh-2022-010720 (PMC9853149; doi:10.1136/bmjgh-2022-010720)
Supplement: Supplementary data [file bmjgh-2022-010720supp002.pdf]

Table S1: Selected indicators of political commitment to UHC and EPHS in the six focus countries

	**Afghanistan**	**Ethiopia**	**Pakistan**	**Somalia**	**Sudan**	**Zanzibar (Tanzania)**
Is the UHC package part of the national SDGs plan and monitoring scheme?	No	Yes	Yes	Yes	Yes	Yes
Were UHC and the package part of the national health vision and strategy?	All yes					
Was there evidence of commitment at the level of parliament in the form of resolution/ decision?	All no					
Was there engagement of the finance and planning sectors during the planning phase?	No	Yes	Yes	Yes	Yes	Yes
